# Supplementary material for: Defined Pig Microbiota with a Potential Protective Effect against Infection with Salmonella Typhimurium
Source: Microorganisms. 2023 Apr 12;11(4):1007. doi: 10.3390/microorganisms11041007 (PMC10146858; doi:10.3390/microorganisms11041007)
Supplement: Supplementary file 1 [file microorganisms-11-01007-s001.zip › microorganisms-2302980-supplementary.pdf]

Supplementary Materials. Identifiacation and functional properties of commensal bacteria isolated from pig and piglets colon and fecal sample.

			Delta log CFU/mL decrease after incubation in								
			pH 3		1.5% bile		Inhibition zones (mm)*				
Strain	MALDI-TOF MS identification	origin	1h	2h	2h	3h	LT2	STM	Agg	Agg%**	Adhesion**
PC1	*Acidomanococcus fermentans*	domestic piglet colon	0.52	0.58	0.52	0.54	6.00 ± 0.00	6.00 ± 0.00	-	nt	1.24 ± 0.75
PC2	*Bacillus licheniformis*	domestic piglet colon	0.75	0.97	0.26	0.44	6.00 ± 0.00	6.00 ± 0.00	-	nt	2.89 ± 0.44
AC1	*Bacillus licheniformis*	domestic pig colon	0.50	1.99	0.00	0.24	6.00 ± 0.00	6.00 ± 0.00	-	nt	0.40 ± 0.16
AC2	*Bacteroides uniformis*	domestic pig colon	0.11	0.14	0.07	0.11	6.00 ± 0.00	6.00 ± 0.00	-	nt	0.21 ± 0.08
PC3	*Bacteroides uniformis*	domestic piglet colon	1.05	1.10	0.17	1.06	6.00 ± 0.00	6.00 ± 0.00	-	nt	0.16 ± 0.06
WP1	*Bifidobacterium boum*	wild pig colon	0.11	0.14	0.11	0.07	9.00 ± 0.00	8.00 ± 0.00	-	nt	0.37 ± 0.11
PC4	*Bifidobacterium boum*	domectic piglet colon	1.05	1.10	0.17	1.06	9.00 ± 0.00	10.00 ± 0.00	-	nt	0.24 ± 0.06
PF1	*Bifidobacterium pseudolongum*	domestic piglet feces	0.00	0.00	0.00	0.02	8.00 ± 0.00	7.67 ± 0.58	-	nt	0.11 ± 0.02
WP2	*Bifidobacterium thermophilum*	wild pig colon	0.11	0.13	0.11	0.27	7.33 ± 0.58	7.00 ± 0.00	-	nt	0.24 ± 0.08
PF2	*Bifidobacterium thermophilum*	domectic piglet feces	0.33	1.03	1.68	1.99	7.67 ± 0.58	13.33 ± 0.58	-	nt	5.98 ± 1.26
AC3	*Clostridium sporogenes*	domestic pig colon	0.41	1.64	2.34	2.66	6.00 ± 0.00	8.33 ± 0.58	-	nt	3.25 ± 0.25
AC4	*Enterococcus durans*	domestic pig colon	0.89	1.16	2.11	2.89	6.00 ± 0.00	6.00 ± 0.00	-	nt	4.42 ± 2.75
WP3	*Enterococcus faecium*	wild pig colon	0.25	0.33	0.64	0.99	9.00 ± 0.00	8.67 ± 0.00	-	nt	0.74 ± 0.36
AC5	*Escherichia coli*	domestic pig colon	0.01	0.32	0.05	0.08	6.00 ± 0.00	6.00 ± 0.00	-	nt	6.13 ± 1.06
AF1	*Escherichia coli*	domestic pig colon	0.02	0.06	0.44	1.44	6.00 ± 0.00	6.00 ± 0.00	-	nt	2.60 ± 0.39
PC5	*Escherichia fergusonii*	domestic piglet colon	0.96	1.52	1.08	1.76	6.00 ± 0.00	6.00 ± 0.00	-	nt	0.38 ± 0.09
PC6	*Lactobacillus amylovorus*	domestic piglet colon	0.03	0.23	1.05	2.01	10.33 ± 0.58	7.00 ± 0.00	+	41.90 ± 2.05	10.37 ± 4.16
WP4	*Lactobacillus porci*	wild pig colon	1.19	1.25	1.11	1.31	8.00 ± 0.00	6.00 ± 0.00	+	77.13 ± 3.35	0.38 ± 0.09
WP5	*Ligilactobacillus ruminis*	wild pig colon	0.69	0.74	1.38	3.69	6.00 ± 0.00	6.00 ± 0.00	-	nt	12.05 ± 1.79
AF2	*Ligilactobacillus ruminis*	domestic pig feces	0.26	0.33	0.69	0.95	8.00 ± 0.00	9.00 ± 0.00	+	78.00 ± 2.95	9.85 ± 1.03
WP6	*Ligilactobacillus salivarius*	wild pig colon	0.09	0.10	0.10	0.10	8.33 ± 0.58	8.67 ± 0.58	-	nt	1.84 ± 0.49
PF3	*Limosilactobacillus mucosae*	wild pig colon	0.19	0.23	0.45	0.79	10.00 ± 0.00	8.67 ± 0.58	+	22.10 ± 0.79	1.14 ± 0.86
WP7	*Limosilactobacillus reuteri*	wild pig colon	0.57	1.03	1.96	2.85	9.67 ± 0.58	7.00 ± 0.00	+	73.83 ± 4.62	2.06 ± 0.84
AF3	*Limosilactobacillus reuteri*	domestic pig feces	0.03	0.13	0.01	0.01	7.00 ± 0.00	9.00 ± 0.00	-	nt	1.92 ± 0.49
PC7	*Paeniclostridium sordellii*	domestic piglet colon	1.15	1.68	2.36	2.98	6.00 ± 0.00	6.00 ± 0.00	-	nt	7.05 ± 0.32

*susceptibility of *Salmonella* strains to cell-free supernatant (diameters are means of three determination ± SD, diameter of the well = 6.00 mm)

**n = 3, mean ± SD

LT2 - *S.* Typhimurium; STM - *S.* Typhimurium

Agg - auto-aggregation (scored positive when clearly visible sand-like particles were formed)

Agg% - auto-aggregation %

nt - not tested
